# Supercritical CO_2_-Mediated Decellularization of Bovine Spinal Cord Meninges: A Comparative Study for Decellularization Performance

**DOI:** 10.1021/acsomega.4c08684

**Published:** 2024-11-25

**Authors:** Eren Ozudogru, Tugce Kurt, Burak Derkus, Ugur Cengiz, Yavuz Emre Arslan

**Affiliations:** 1 Regenerative Biomaterials Laboratory, Department of Bioengineering, Faculty of Engineering, Canakkale Onsekiz Mart University, Canakkale 17100, Turkey; 2 Stem Cell Research Laboratory, Department of Chemistry, Faculty of Science, Ankara University, Ankara 06560, Turkey; 3 Surface Science Research Laboratory, Department of Chemical Engineering, Faculty of Engineering, Canakkale Onsekiz Mart University, Canakkale 17020, Turkey

## Abstract

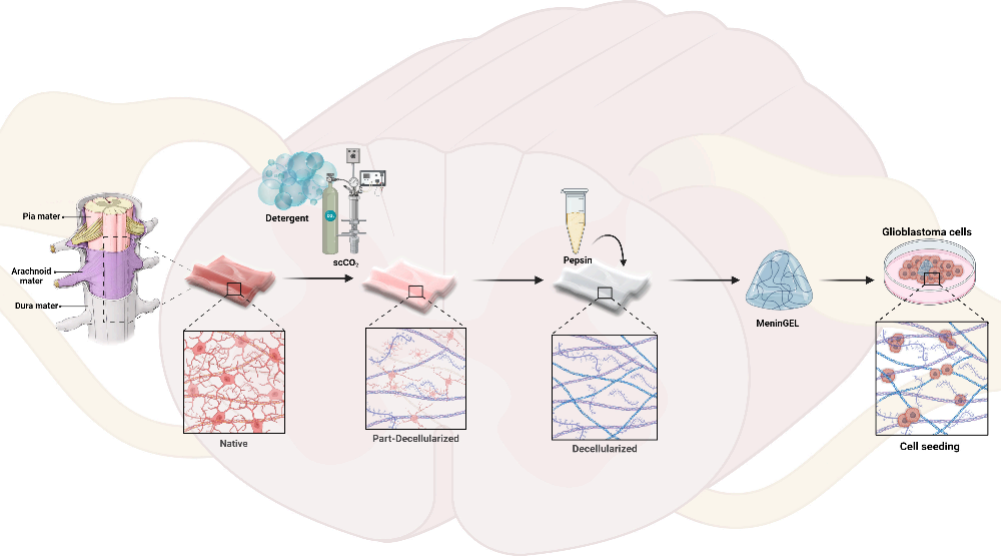

The extracellular matrix (ECM) of spinal meninge tissue closely resembles the wealthy ECM content of the brain and spinal cord. The ECM is typically acquired through the process of decellularizing tissues. Nevertheless, the decellularization process of the brain and spinal cord is challenging due to their high-fat content, in contrast to the spinal meninges. Hence, bovine spinal cord meninges offer a promising source to produce ECM-based scaffolds, thanks to their abundance, accessibility, and ease of decellularization for neural tissue engineering. However, most decellularization techniques involve disruptive chemicals and repetitive rinsing processes, which could lead to drastic modifications in the tissue ultrastructure and a loss of mechanical stability. Over the past decade, supercritical fluid technology has experienced considerable advancements in fabricating biomaterials with its applications spreading out to tissue engineering to tackle the complications mentioned above. Supercritical carbon-dioxide (scCO_2_)-based decellularization procedures especially offer a significant advantage over classical decellularization techniques, enabling the preservation of extracellular matrix components and structures. In this study, we decellularized the bovine spinal cord meninges by seven different methods. To identify the most effective approach, the decellularized matrices were characterized by dsDNA, collagen, and glycosaminoglycan contents and histological analyses. Moreover, the mechanical properties of the hydrogels produced from the decellularized matrices were evaluated. The novel scCO_2_-based treatment was completed in a shorter time than the conventional method (3 versus 7 days) while maintaining the structural and mechanical integrity of the tissue. Additionally, all hydrogels derived from scCO_2_-decellularized matrices demonstrated high cell viability and biocompatibility in a cell culture. The current study suggests a rapid, effective, and detergent-free scCO_2_-assisting decellularization protocol for clinical tissue engineering applications.

## 1 Introduction

The prevalence of chronic diseases worldwide has resulted in a significant increase in annual deaths due to the lack of available transplant organs.^1^ One potential solution to this problem is the production of new artificial tissues and organs for implantation through tissue engineering. The most popular tissue engineering strategy uses cross-linked porous polymeric materials that mimic the natural tissue environment.^2^ However, the degradation of implanted synthetic polymers reduces the pH at the regeneration site, and this causes cell cytotoxicity, inflammation, and tissue damage over time.^3^ Hence, highly biocompatible natural materials that enable cell attachment and proliferation and thereby guide tissue reconstruction have been desired. The decellularization process aims to preserve all ECM treasures and minimize the risk of immune rejection in allogeneic or xenogeneic applications while eliminating cellular units from tissues.^4^ Thus, decellularized materials closely imitate the dynamic, functional, and bioactive structure of native tissue by inheriting their collagen, elastin, sulfated glycosaminoglycans (sGAGs), growth factors, and cytokines.^5^ Additionally, decellularized ECM has been noted to provoke an anti-inflammatory immune response.^6^ Decellularized materials are fabricated with a sequence of tissue-specific decellularization processes that involves lysing the cell membranes, solubilizing the cytoplasmic and nuclear ingredients, and removing the cellular remnants from the ECM.^7^ To achieve these functional biomaterials, decellularization is employed following various methodologies, such as freeze–thaw cycles and physical, chemical, and enzymatic treatments. Furthermore, detergents like sodium dodecyl sulfate (SDS), sodium deoxycholate, and Triton X-100 are the most frequently used for decellularization.^8^ Nonetheless, these detergents generally involve aggressive conditions that denature proteins and disrupt sGAG, growth factors, and ECM ultrastructure.^9,10^ Moreover, the residuals of these detergents may persist within the tissue, potentially leading to cytotoxicity and adverse effects.^11^ Thereof, the necessity for clinically applicable techniques has emerged to decellularize tissues successfully without using harsh chemicals or solvents for prolonged periods. Supercritical carbon dioxide (scCO_2_) is regarded as an environmentally friendly alternative to conventional chemical techniques in accordance with the principles of green chemistry.^12^ scCO_2_ is a promising tool for decellularization thanks to its unique properties, including low surface tension, high diffusivity, and nontoxicity.^13−15^ In addition, scCO_2_ exhibits both liquid and gas-like properties,^13,14^ allowing it to penetrate tissues and effectively remove cellular components without significantly damaging the ECM. Also, it has favorable solvent properties and a slight critical temperature (31.06 °C), making it appropriate for use at physiological temperatures.^16^ Ultimately, using scCO_2_ reduces the necessity for downstream processing and purification steps, thereby enhancing the efficiency and sustainability of the process.^17^ Many tissues, including the aorta,^6^ cornea,^18^ bone,^19^ and adipose tissue,^20^ have been reported to be effectively decellularized with scCO_2_. The spinal meninges are composed of three distinct layers: the dura mater, the arachnoid mater, and the pia mater. These layers encircle the spinal cord, thus ensuring the maintenance of tissue homeostasis and protecting it from external injuries. Additionally, its ECM presents a variety of proteins that play vital roles in nerve development, survival, and regeneration, such as GAGs; collagen types I, III, IV, V, and VI; perlecan; fibronectin; laminins; galectin; and neurofascin.^21,22^

In this study, we aimed to optimize a fast, effective, and detergent-free scCO_2_ decellularization method for spinal cord meninges. To achieve this, we employed seven different protocols, utilizing a traditional detergent-based technique as a control. As a proof of concept, we investigated cell removal and ECM damage in detail via biochemical, histological, and mechanical analyses. The cytocompatibility of glioblastoma cells on decellularized spinal meninges was also approved by cyto-viability tests. To the authors’ knowledge, the combination of scCO_2_/enzymatic methods supplies highly effective decellularization of tissues.

## 2 Materials and Methods

### 2.1 Preparation of Bovine Spinal Cord Meninges

The bovine spinal cord meninges were acquired from a local abattoir in Lapseki, Çanakkale. Initially, the spinal meninges were separated from the spinal cords and rinsed with water to remove impurities, such as blood and fats. Afterward, the tissues were cut into approximately 1 cm × 1 cm pieces for decellularization. Unless otherwise stated, all chemicals and reagents were purchased from Merck.

### 2.2 Decellularization of Spinal Cord Meninges with scCO_2_

Three different groups and seven sets of experiments were designed to identify the optimal parameters for the decellularization of bovine spinal meninges by using scCO_2_. Each set varied in specific conditions to comprehensively evaluate the impacts of pressure and enzymatic treatment methods. Approximately 2.2 g (10 pieces) of wet meninges tissues was used for each set. Based on the literature research, 70% ethanol was used as a cosolvent in all experiments performed with scCO_2,_ which were carried out at 37 °C.^23^ A mixture consisting of 10 meninge tissues was placed into a 100 mL stainless-steel reactor equipped with a mechanical stirrer. The pressure and temperature within the reactor were controlled by using a PLC controller and a pressure gauge. To remove air from the system, CO_2_ was introduced via a Maximator M-37 pump, pressurizing the reactor to 65 bar while simultaneously heating it to 37 °C with continuous stirring at 250 rpm.^24,25^ The system setup is given in Figure S1. The reactor was then pressurized further to the required level with CO_2_ at 37 °C, and the reaction was allowed to proceed for 4 h. After completion, CO_2_ was released, and the decellularized meninges were retrieved in a methanol solution. All groups are categorized as follows.

#### Group I: Evaluation of Pressure

180, 200, and 220 bar(e−) refer to tissues exposed to scCO_2_ at 180, 200, and 220 bar pressure, respectively, 70% ethanol cosolvent, 37 °C temperature, and 200 rpm agitation for 4 h (“e–″ denotes the absence of enzymatic treatment).

#### Group II: Evaluation of Enzymatic Treatment

180, 200, and 220 bar(e+) refer to group I conditions at 180, 200, and 220 bar, respectively, but include enzymatic treatment and decontamination processes after scCO_2_ exposure (“e+″ denotes the presence of enzymatic treatment).

#### Group III: Conventional Detergent Method (Control)

Detergent(e+) refers to the decellularization of spinal meninges via 1% Triton X-100 solution, enzymatic treatment, and decontamination, as previously described by our group.^26^

These varied experimental conditions were systematically assessed to elucidate the most effective protocol for achieving successful decellularization while maintaining tissue structure and integrity and minimizing antigenicity.

After exposure to scCO_2_, the tissues were rinsed with Milli-Q water for several rounds to purify them from ethanol residues. Moreover, meninges were incubated in 40 μg/mL DNase (≥400 Kunitz units/mg protein, DN25, Merck, Germany) and 20 μg/mL RNase (50–100 Kunitz units/mg protein, R5503, Merck, Germany) solutions prepared in 10 mM MgCl and 50 mM Trizma hydrochloride buffer (pH 7.5) at 37.5 °C for 24 h in Incu-Shaker (Benchmark, USA) to eliminate DNA and RNA remnants from the tissues. Following the enzymatic treatment, the meninges were rinsed and decontaminated with 4% ethanol and 0.1% peracetic acid solution for 5 h in RT under rotation. Finally, the tissues were rinsed with ultrapure water for overnight.

### 2.3 Biochemical Analyses

The effectiveness of the decellularization process was confirmed by double-stranded DNA (dsDNA), hydroxyproline (HYP), and sGAG content analyses, agarose gel electrophoresis, and sodium dodecyl sulfate-polyacrylamide gel electrophoresis (SDS-PAGE). In this regard, dsDNA was isolated from native and decellularized tissue samples using the PureLink Genomic DNA purification kit (Thermo Fisher Scientific, USA) following the kit directives. The dsDNA content was determined with a Qubit 4 Fluorometer (Invitrogen, Thermo Fisher Scientific, USA) using the Qubit 1× dsDNA Broad Range (BR) Assay Kit for native tissue and group I samples and the Qubit 1× dsDNA High Sensitivity (HS) Assay Kit for group II and group III samples according to the kit protocol. Furthermore, HYP content was detected by the Elabscience HYP colorimetric assay kit (E-BC-K062-S) applying the manufacturer’s instructions. Ultimately, sGAG content was quantified using the dimethylmethylene blue (DMMB) assay, SDS-PAGE was performed according to the Laemmli method, and agarose gel electrophoresis was conducted as previously described by our team.^26^ The band intensity on the agarose gel was quantified by densitometric analysis in ImageJ (version 1.53t, National Institutes of Health, USA). The percentage band density of the samples was calculated by assuming the band density of the native tissue to be 100%.

### 2.4 Histological Analyses

Hematoxylin and eosin (H&E), Sirius red, and Alcian blue stainings were subjected to assess the efficacy of the decellularization process and its impact on tissue architecture. To prepare tissues for staining, the samples were submerged in a 10% neutral buffered formalin for 2 days and then dehydrated through a series of alcohols and embedded in paraffin. Subsequently, specimens were sectioned into 3–5 μm thick with a microtome. The routine H&E, Sirius red, and Alcian blue staining techniques were accomplished.^27,28^ For histological evaluation, images were acquired using a light microscope equipped with an Axiocam 105 color camera (Zeiss, Germany). The intensity of the Sirius red and Alcian blue stainings was evaluated using ImageJ software (version 1.53t, National Institutes of Health, USA).

DAPI (4′,6-diamidino-2-phenylindole, dihydrochloride) staining was applied to the sections to confirm the absence of cellular DNA in the tissues postdecellularization. For this purpose, the sections were treated with 0.1% (v/v) DAPI.^29^ Finally, DAPI images were investigated by fluorescence microscopy (Leica DM IL, Germany) at 358 and 461 nm (excitation/emission).

### 2.5 Scanning Electron Microscopy

Field-emission scanning electron microscopy (FE-SEM JFM-7100F EDS, JEOL, Japan) was employed to characterize the surface morphologies of native and decellularized spinal meninges. For this analysis, the tissues were initially immersed in a 2.5% glutaraldehyde solution prepared with PBS (v/v) at pH 7.2–7.4 for 24 h. Afterward, each sample was dehydrated for 5 min in a series of solutions containing 50, 70, 80, 90, 95, and 100% ethanol, respectively. Subsequently, the samples were allowed to dry fully at room temperature and sputter-coated (SC7620, Mini Sputter Coater, England) with a thin layer of gold–palladium for 90 s. SEM micrographs were obtained at 10 kV at varying degrees of magnification in a high vacuum.

### 2.6 Preparation of Hydrogel

First, the decellularized spinal cord meninges (dSCM) were homogenized. Thereafter, the emulsion was frozen at <−20 °C and then freeze-dried overnight. The lyophilized dSCMs (5, 7.5, and 10 mg/mL) were digested with 1 mg/mL pepsin (Sigma-77160) in 0.01 M HCI for 72 h at RT under rotation. The obtained pregel was neutralized with 7.5% sodium bicarbonate at a 10:1 (v/v) ratio in an ice bath and incubated at 37 °C for 1 h to cross-link collagen fibrils. After this process, the hydrogel obtained from the detergent-treated meninges was named MeninGEL. The hydrogels achieved from scCO_2_ at 180, 200, and 220 bar pressures and then nuclear enzyme-treated meninges were called MeninGEL-180 bar(e+), MeninGEL-200 bar(e+), and MeninGEL-220 bar(e+), respectively.

### 2.7 Mechanical Properties and Rheological Analysis

The mechanical characteristics of the hydrogels were assessed through compression tests and rheology analysis. Compression analysis was executed by CellScale Biomaterials Testing (UniVert, Canada), using a 10 N load cell at 0.05 mm/s. Also, rheological evaluations were conducted with a Discovery Hybrid Rheometer (TA Instruments) using a smooth parallel plate (φ = 20 mm, gap 0.9 ± 0.1 mm). The hydrogels’ LVR data were acquired via oscillatory strain sweep in the 0.01–100% strain range and at a frequency of 1 Hz. Lastly, the frequency sweep was fulfilled, ranging from 0.1 to 100 Hz and at 37 °C.

### 2.8 Attenuated Total Reflectance Fourier Transform Infrared Spectroscopy (ATR-FTIR)

A Nicolet iS50 Flex Gold Infrared Spectrometer was used to detect differences in chemical bonds between native tissue and scCO_2_-decellularized meninges. Each IR spectrum was scanned 64 times and collected in the 4000–400 cm^–1^ wavenumber range with a resolution of 16 cm^–1^ at RT.

### 2.9 Thermogravimetric Analysis (TGA)

Thermogravimetric behaviors of native and decellularized meninge tissues were ascertained with a PerkinElmer TGA 8000. The analysis was conducted under a nitrogen gas flow of 15 mL/min and 10 °C/min rate between 30 and 800 °C.

### 2.10 Cytocompatibility and Cell Viability

Live/dead and 2,3-bis(2-methoxy-4-nitro-5-sulfophenyl)-2*H*-tetrazolium-5-carboxanilide (XTT, Biological Industries, USA) assays were subjected to assessment of the cellular adhesion, survival, proliferation, and morphology of glioblastoma cells (U-87 MG, ATCC number: HTB-14) on MeninGELs. The primary glioblastoma cells were initially cultured according to the provider’s procedures. To conduct the live/dead assay, MeninGELs were placed in a 48-well plate, and glioblastoma cells (10,000 cells per gel) were cultured on the hydrogels under standard culture conditions (at 37 °C, 5% CO_2_, and 95% relative humidity) for 2 and 5 days. Subsequently, the cells were stained with Calcein-AM/EthD-1, and the cell viability was evaluated using fluorescence microscopy. To gain further vision, XTT analysis was conducted to ascertain the proliferation of cells on MeninGELs. First, MeninGELs were prepared in a 48-well plate, and glioblastoma cells were seeded at a density of 10,000 cells/hydrogel at 37 °C, 5% CO_2_, and 95% relative humidity for 2 and 5 days. Then, the hydrogels were washed, and the XTT reagent was applied to them for 4 h. Eventually, the cell viability was calculated by measuring the absorbance values at a wavelength of 490 nm using a microplate spectrophotometer. All experiments were conducted in triplicate.

### 2.11 Statistical Analysis

The analysis results were assessed using Microsoft 365 Apps for enterprise Excel and reported as means with standard deviation. Statistical significance between the two related groups was determined using Tukey’s one-way analysis of variance (ANOVA) test with OriginPro 2024 (v10.1.0.170, Learning Edition, OriginLab Corporation, Massachusetts, USA). Significant data points were highlighted with an asterisk (*), and the threshold for statistical significance was appointed at a *p*-value of less than 0.05.

## 3 Results and Discussion

### 3.1 Evaluation of the Decellularization Process

**Figure 1:**
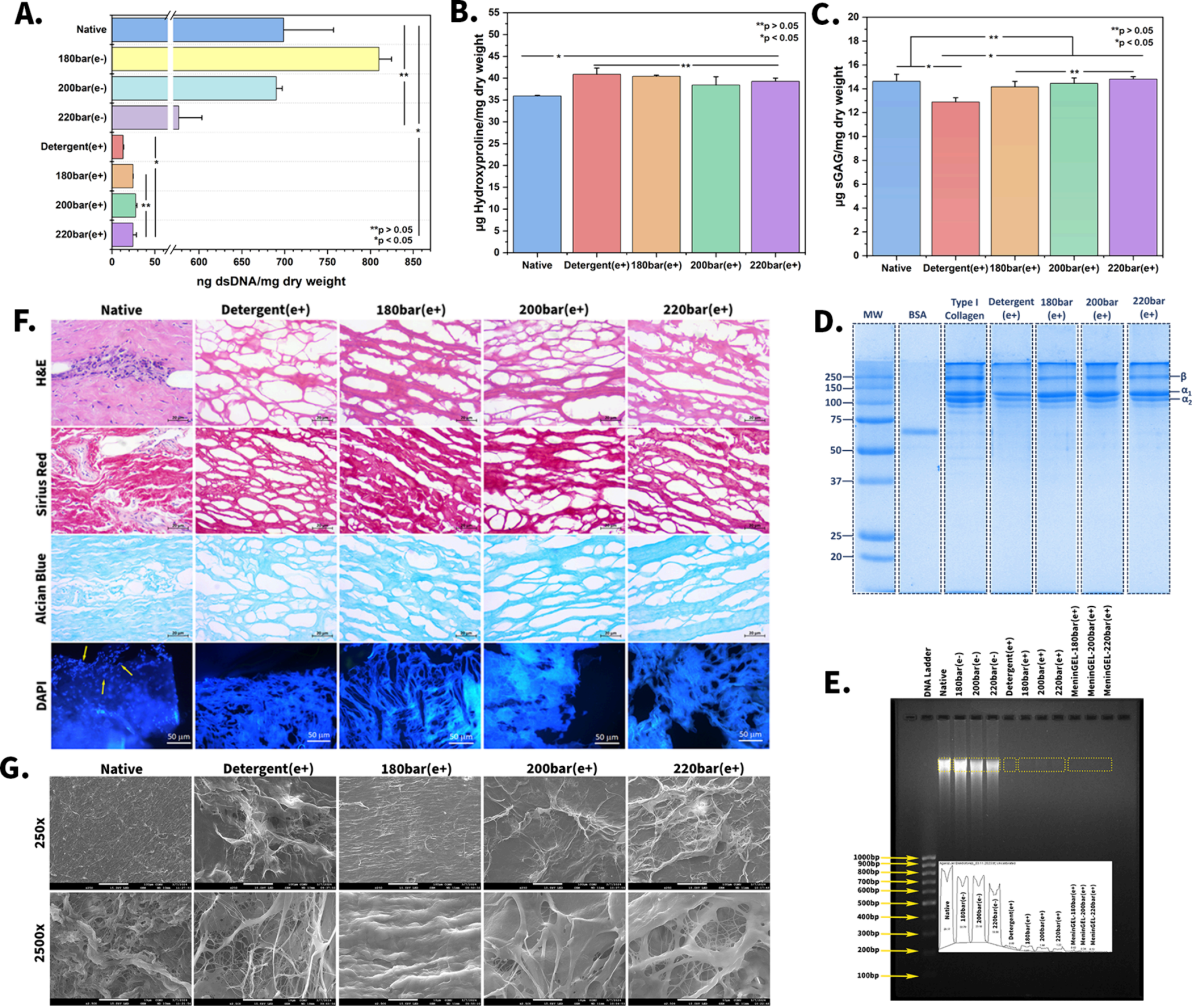
Biochemical characterizations and SEM analysis. dsDNA content (A), hydroxyproline content (B), glycosaminoglycan content (C), SDS-PAGE analysis (D), agarose gel electrophoresis (E), histology (F), and characterization of surface morphology (G) of native and decellularized spinal cord meninges by SEM analysis.

The primary objective of decellularization is to effectively eliminate immune-triggering components (such as cellular particles, nuclear materials, and alpha-Gal epitopes) while preserving the intrinsic structure of the tissue, including sGAG, collagen, and other essential ingredients.^30^ Stephen Badylak, a pioneer in the field of decellularization, and his colleagues proposed a set of criteria for the amount of residual dsDNA in decellularized tissue. According to their proposal, the amount of dsDNA in the decellularized tissue should be less than 50 ng per mg of extracellular matrix (ECM) dry weight. The length of the DNA fragment should be less than 200 bp (base pairs), and there should be no visible nuclear material in tissue sections stained with DAPI or H&E to eliminate the risk of an immune response.^31^ The majority of scientists globally have accepted these criteria as appropriate for decellularization.^32,33^ Accordingly, to ascertain the efficacy of the decellularization techniques, a series of analyses were conducted, involving assessments of dsDNA, HYP, and sGAG content; histological examinations using H&E, DAPI, Sirius red, and Alcian blue stains; agarose gel electrophoresis; and SDS-PAGE analyses. The dsDNA content analysis revealed that the dsDNA contents of the native tissue, 180 bar(e−), 200 bar(e−), 220 bar(e−), detergent(e+), 180 bar(e+), 200 bar(e+), and 220 bar(e+), were found to be 698.74 ± 58.24, 810.01 ± 14.7, 690.2 ± 6.98, 576.47 ± 26.93, 12.97 ± 0.7, 24.44 ± 0.09, 27.69 ± 1.44, and 24.45 ± 4.05 ng/mg dry weight, respectively (*n* = 3; Figure 1A). The data indicate that the dsDNA content was reduced by approximately 98.14, 96.5, 96.04, 96.5, and 96.5% in detergent(e+), 180 bar(e+), 200 bar(e+), and 220 bar(e+) samples, respectively. As seen in Figure 1A, the dsDNA values of 180 bar (e−), 200 bar (e−), and 220 bar (e−) samples were higher than the level required (<50 ng/mg dry weight) for successful decellularization. Furthermore, there was no significant difference in the dsDNA content between the native tissue, 180 bar (e−), 200 bar (e−), and 220 bar (e−) samples (*n* = 3; *p* > 0.05; ANOVA, Figure 1A). Nevertheless, the dsDNA content values of all samples (180, 200, and 220 bar(e+)) treated with DNase/RNase enzyme and scCO_2_ application under 70% ethanol and 180, 200, and 220 bar conditions were below 50 ng/mg dry weight. Moreover, there was no statistically significant difference between these values (*n* = 3; *p* > 0.05; ANOVA, Figure 1A). As determined by dsDNA content analysis, the traditional method—involving 1% Triton X-100 and DNase/RNase—removed the maximum nuclear material from the tissue. Consequently, without the application of additional enzyme treatment, scCO_2_ application under the aforementioned conditions was not sufficient for decellularization. These findings align with those of Sawada et al., who previously observed that scCO_2_ alone, without additional treatment, is ineffective in decellularization.^16^ The actual images of the native and scCO_2_ treatment tissues are given in Figure S2.

ECMs predominantly comprise collagens and sGAG.^34,35^ The quantities of HYP and collagen are directly related, as HYP constitutes approximately 14% of collagen, the most prevalent protein in ECM.^21^ HYP content analysis was carried out to evaluate the effect of decellularization on the collagen content of the tissue. According to this analysis, the HYP contents of native, detergent(e+), 180 bar(e+), 200 bar(e+), and 220 bar(e+) samples were 35.94 ± 0.15, 40.88 ± 1.43, 40.46 ± 0.24, 38.43 ± 1.89, and 39.28 ± 0.74 μg/mg dry weight, respectively (*n* = 3, Figure 1B). As shown in Figure 1B, there is no significant difference in HYP contents between the detergent(e+) and scCO_2_ decellularization methods (*n* = 3, ANOVA, *p* > 0.05). However, the HYP contents of all decellularized samples increased following decellularization, with a significant difference between them and the native tissue (*n* = 3, ANOVA, *p* < 0.05, Figure 1B). This phenomenon may be attributed to the substantial reduction in volume that occurs during the decellularization process because the decellularized tissue exhibited a markedly elevated concentration of HYP per unit of dry weight in comparison to the native tissue.^36^

sGAG (e.g., hyaluronic acid, chondroitin sulfate, dermatan sulfate, keratin sulfate, heparin sulfate, and heparin) are composed of repeating disaccharide units of uronic acid and amino sugar and are essential for the biomechanical properties of tissues due to their significant water-binding capacity. sGAG chains occupy most of the extracellular spaces and provide mechanical support to the tissue. Additionally, they facilitate the rapid diffusion of water-soluble molecules and the migration of cells.^37^ The sGAG amounts of native tissue, detergent(e+), 180 bar(e+), 200 bar(e+), and 220 bar(e+) samples were determined to be 14.64 ± 0.58, 12.89 ± 0.36, 14.16 ± 0.46, 14.45 ± 0.46, and 14.82 ± 0.21 μg/mg dry weight, respectively (*n* = 3, Figure 1C). The results of the sGAG content analysis revealed no statistically significant difference between the sGAG content analysis of native tissue and the 180 bar(e+), 200 bar(e+), and 220 bar(e+) samples (*n* = 3, ANOVA, *p* > 0.05, Figure 1C). The sGAG content of the detergent(e+) sample exhibited a notable decline compared to native tissue and tissues decellularized with scCO_2_ (*n* = 3, ANOVA, *P* < 0.05, Figure 1C). The degradation or denaturation of sGAG may occur due to the type and concentration of detergent utilized. This can lead to loss of biological activity and functionality of the bioscaffold.^4^ Nonionic detergents, such as Triton X-100, have the potential to strongly disrupt lipid–lipid and lipid–protein bonds yet have less effect on the protein–protein interaction. The effectiveness of these detergents depends on the tissue undergoing the decellularization process.^10^ Consequently, although Triton X-100 detergent is an effective agent for the removal of nuclear materials such as DNA/RNA from bovine spinal meninge tissue, it may have resulted in a reduction in sGAG in the tissue. In addition, the results indicate no significant difference in the effects of 180, 200, and 220 bar pressures in the scCO_2_ treatment on dsDNA, HYP, and sGAG content.

Collagen is the most abundant structural protein in humans and other mammals. Its primary function is to provide structural support to tissues. Although type I collagen represents only one of the more than 20 different structural forms of collagen, it is the primary component of numerous tissues and comprises approximately 90% of the total collagen content in the human body.^38,39^ Hence, commercial collagen type I is employed as a validation agent for the identification of proteins by the SDS-PAGE analysis of decellularized biomaterials. Figure 1D illustrates the presence of β, γ, α_1_, and α_2_ collagen chains in meninges tissue decellularized by different methods.^26,40^

The effectiveness of decellularization might be assessed by comparing the intensity of the DNA bands on the agarose gel. A less intense band in the decellularized tissue sample indicates that a greater quantity of DNA has been removed, thereby confirming the effectiveness of the decellularization process. To support this hypothesis, densitometric analysis was performed on agarose gel bands. The band intensities of native, 180 bar(e−), 200 bar(e−), 220 bar(e−), 180 bar(e+), 200 bar(e+), 220 bar(e+), and detergent(e+) samples were computed as 100, 80.9, 82, 69.86, 6.43, 5.11, 4.37, and 2.84%, respectively (Figure 1E). These data demonstrated that approximately 93% or more dsDNA was removed from enzyme-treated tissues. In addition, band intensities of the MeninGEL-180 bar(e+), MeninGEL-200 bar(e+), and MeninGEL-220 bar(e+) samples were 1.85, 1.21, and 0.43%, respectively (Figure 1E). Considering these data, pepsinization treatment resulted in an average of 5% more dsDNA removal on the same samples, and no visible dsDNA bands were detected on agarose gel. This situation may be associated with the acidic medium in which the pepsinization process was conducted. Although the pepsin enzyme has no known effect on the DNA molecule, the acidic medium may cause denaturation by breaking the hydrogen bonds between complementary base pairs.^41^ The agarose gel electrophoresis results are consistent with those of the dsDNA content analysis.

H&E, Sirius red, and Alcian blue staining analyses play a vital role in their characterization by providing valuable information on decellularized tissue structure and ECM composition.^40^ H&E staining serves to verify the efficacy of the decellularization process. After decellularization, tissues or biomaterials should ideally be devoid of cellular debris. This analysis allows a qualitative assessment of nuclear residues.^8^ Also, Sirius red is a selective dye that binds specifically to collagen fibers, an essential structural component of ECM. Thus, Sirius red enables the visualization of the spatial distribution and arrangement of collagen fibers.^42^ Alcian blue specifically stains acidic polysaccharides such as sGAG. Namely, the intensity of the Alcian blue supplies a quantitative estimate of the overall sGAG content.^40^ Finally, DAPI is a fluorescent dye that binds specifically to DNA. DAPI staining, similar to H&E, confirms the successful removal of nuclear components throughout the decellularization process.^4^ In both H&E and DAPI stainings, cell nuclei in the native tissue were clearly visible, whereas no cellular material was found in the decellularized tissues (Figure 1F). Histological images demonstrate that detergent(e+), 180 bar(e+), 200 bar(e+), and 220 bar(e+) methods employed are effective in removing the cells. Figure 1F illustrates the porous structure of the spinal meninges tissue. Furthermore, the Sirius red staining revealed the presence of a dense collagen network in the samples. The Sirius red staining intensity of native, 180 bar(e+), 200 bar(e+), 220 bar(e+), and detergent(e+) samples were calculated as 76.43, 73.55, 58.56, 55.04, and 59.03%, respectively. The intensity of Sirius red in the 180 bar(e+) sample is similar to that of the native tissue and more intense than that of the detergent(e+), 200 bar(e+), and 220 bar(e+) samples (Figure S3, Supporting Information). Furthermore, the Alcian blue staining intensities of native, 180 bar(e+), 200 bar(e+), 220 bar(e+), and detergent(e+) samples were determined as 83.08, 64.45, 60.49, 57.92, and 52.48%, respectively (Figure S3, Supporting Information). According to these results, the 180 bar(e+) sample demonstrates the most analogous characteristics to native tissue in Alcian blue staining intensity, whereas the detergent(e+) sample exhibits the lowest intensity among all samples. Consequently, it can be declared that Triton X-100 and 220 bar pressure negatively affect both collagen fibers and sGAG. The histologic evaluations were in agreement with the results of the biochemical analyses.

### 3.2 The Surface Morphology of Decellularized Tissues

Surface imaging with SEM revealed the histoarchitecture of the native tissue and decellularized spinal meninges. The SEM micrographs showed that all of the samples maintained their tissue integrity after the decellularization process. Figure 1G illustrates the ordered, dense, and high-strength collagen fibers of the native spinal meninges tissue.^26^ As depicted in Figure 1G, the ordered and dense collagen fiber structures of the detergent(e+), 200 bar(e+), and 220 bar(e+) samples were disrupted. Conversely, the 180 bar(e+) sample maintained the architectural integrity and microtopographic features of the spinal meninge tissue (Figure 1G). In conclusion, SEM micrographs revealed the Triton X-100 detergent, and the pressures of 200 and 220 bar disrupted the hierarchical tissue organization of spinal meninges.

### 3.3 Physicochemical Characterizations

**Figure 2:**
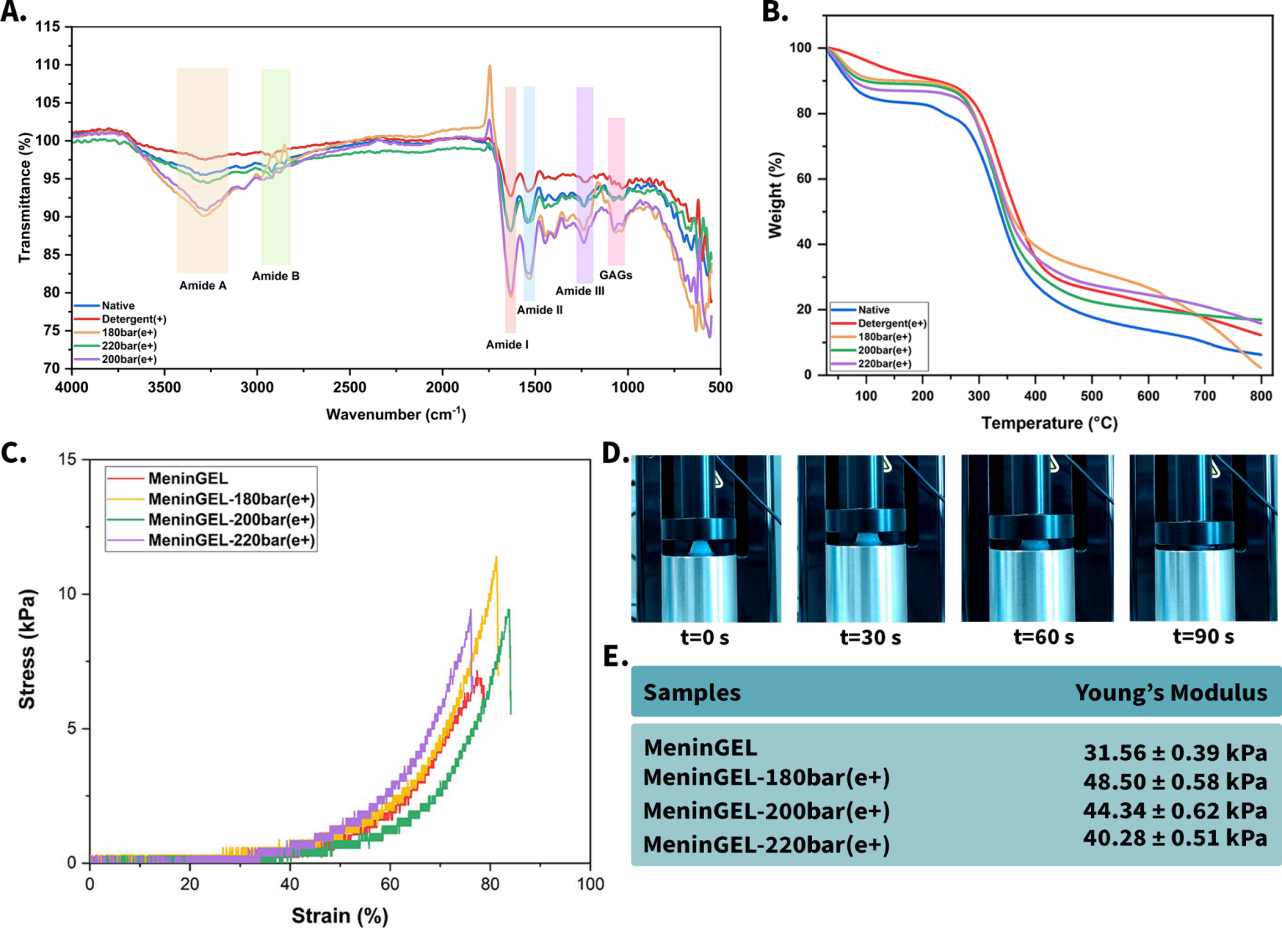
Physicochemical and mechanical characterizations. Chemical bond structures of native and decellularized spinal cord meninges via ATR-FTIR (A), thermal degradation kinetics of native and decellularized membranes by TGA (B), mechanical behaviors of MeninGEL hydrogels (C), actual images of MeninGEL during the compression test (D), and elastic moduli table of MeninGEL (E).

ATR-FTIR analysis was conducted to compare the chemical bond variances between native and decellularized tissues. As seen in Figure 2A, in all groups, the sharp peaks at 3307 cm^–1^ (amide A) and 2927 cm^–1^ (amide B) were attributed to the O–H and N–H stretching of phospholipid, glycolipid, and fatty acids, respectively.^43^ Also, amide I, II, and III bonds are designated as collagen fingerprints. The intense peaks at 1654 cm^–1^ represent amide I (C=O stretching). Furthermore, the sharp peaks at 1556 cm^–^^1^ represent amide II (N–H bending and C–N stretching), while the sharp peaks at 1238 cm^–1^ represent amide III (C–N stretching and N–H bending) bonds.^44,45^ The wavenumber difference between the amide I and II peaks was less than 100 cm^–1^. This indicates the preservation of the triple-helix structure of collagen.^46^ At last, the absorbances at 1048 cm^–1^ were related to the core of sGAG.^47^

TGA is a powerful analytical technique used to investigate the thermal stability and composition of biomaterials.^48^ In this analysis, total organic mass loss for all samples occurred in the temperature range of 50–600 °C (Figure 2B). Two sharp mass loss curves can be seen in the graph. The first mass loss curve in the thermograms between 50 and 100 °C shows the samples’ total water content loss, corresponding to approximately 10–15% of their mass.^49^ Furthermore, approximately 70% of the sample mass was degraded between 250 and 500 °C, corresponding to the thermal degradation of collagen and other noncollagenous proteins.^50^ The TGA thermograms overlap with our previous studies and those of collagen in the literature.^26,51^

### 3.4 Mechanical Behaviors of MeninGEL Hydrogels

The mechanical properties of biomaterials are crucial in determining their potency in clinical settings. These properties profoundly influence their biodegradability and the signaling pathways that regulate scaffold–cell interactions. In other words, mechanical properties affect the ultimate repair performance of bioimplants.^52^ Compression analysis was conducted to ascertain the mechanical properties of the hydrogels. The maximum compressive strengths of MeninGEL, MeninGEL-180 bar(e+), MeninGEL-200 bar(e+), and MeninGEL-220 bar(e+) were 7.16, 11.4, 9.44, and 9.15 kPa, respectively (Figure 2C–D). Their Young’s moduli were also calculated to be 31.56 ± 0.39, 48.50 ± 0.58, 44.34 ± 0.62, and 40.28 ± 0.51 kPa, respectively (*n* = 3, Figure 2E). The graphs related to the calculations are given in Figure S4A–D. These findings could be explained by considering the known mechanisms of how detergents and supercritical fluids interact with cells and proteins. Most detergents, such as Triton X-100, have the capacity to damage protein–protein structures in the ECM.^9^ In contrast, scCO_2_ is able to penetrate tissue and remove cells thanks to its both liquid and gas-like properties without affecting protein–protein interactions.^7^ The compression analysis results, in accordance with the literature, show that exposure to detergents negatively affects the strength of biomaterials.^10^

### 3.5 Rheological Assessment

**Figure 3:**
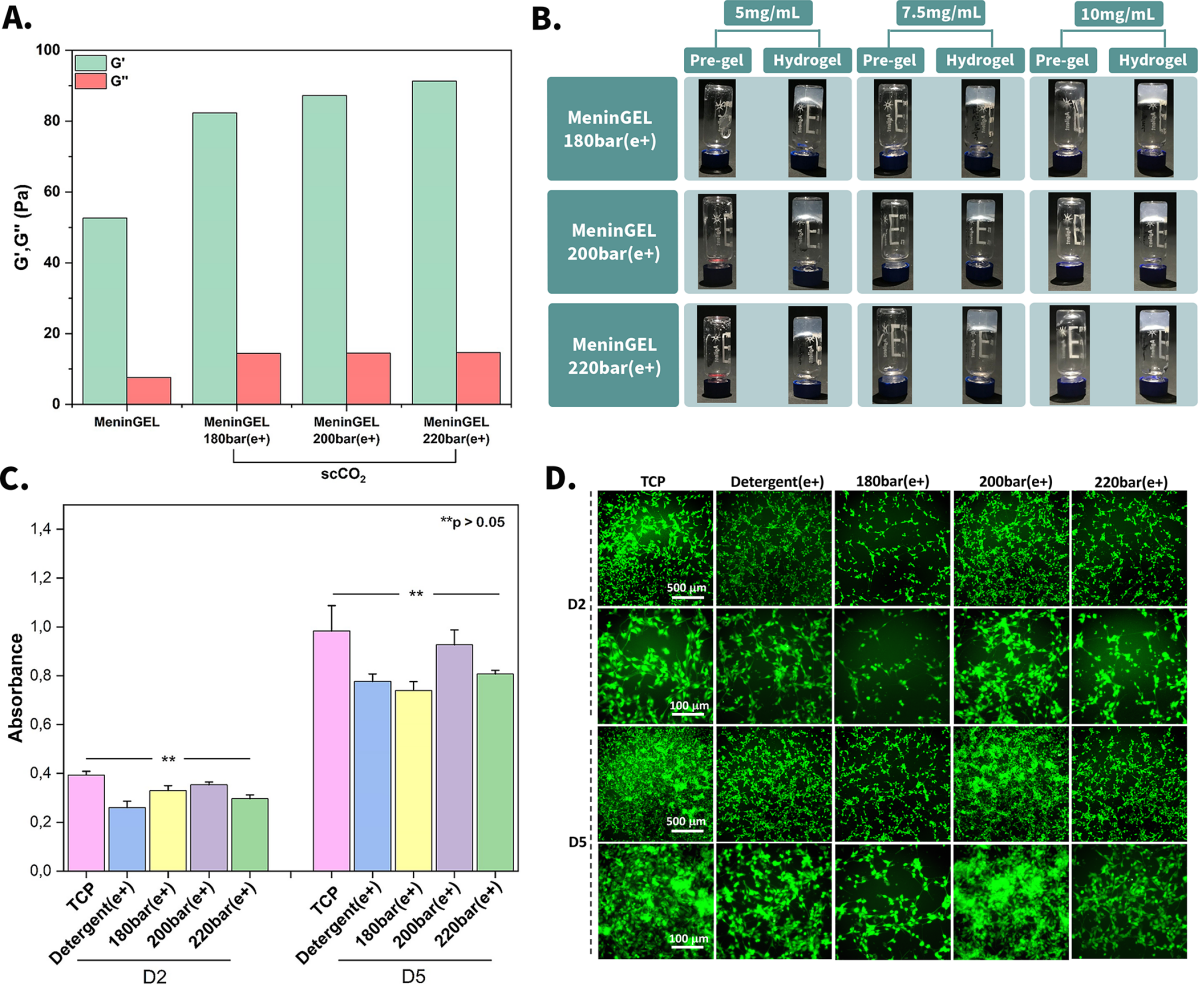
Gel properties and biological evaluations. Rheology analysis (A), gelation performance of MeninGEL (B), biocompatibility analysis of MeninGEL hydrogels with XTT analysis (C), and cell viability of glioblastoma cells on MeninGEL by live/dead assay (D), scale bars indicate 500 and 100 μm.

In rheological analysis, *G*′ represents the elastic or storage modulus. It measures the ability of a material to store and recover energy when it is subjected to deformation. Moreover, a higher *G*′ value indicates a more elastic response, which signifies that the material can more efficiently resist deformation. Materials with high *G*′ values are often described as more rigid-like or elastic. *G*″ also represents the viscous or loss modulus. *G*″ provides insight into the capacity of a material to dissipate energy in the form of heat when subjected to deformation. In addition, a higher *G*″ value expresses a more viscous response, meaning the material has greater resistance to flow.^53^ The storage modulus of MeninGEL-220 bar(e+) was found to be 1.05-, 1.11-, and 1.73-fold that of MeninGEL-200 bar(e+), MeninGEL-180 bar(e+), and MeninGEL, respectively. Similarly, the loss modulus of MeninGEL-220 bar(e+) was 1.01, 1.02, and 1.9 times that of MeninGEL-200 bar(e+), MeninGEL-180 bar(e+), and MeninGEL, respectively (Figure 3A). The results demonstrate that the scCO_2_-decellularized samples exhibit a more elastic behavior compared to MeninGEL. In both compression and rheological analyses, MeninGEL displayed a lower mechanical performance than the others. In light of these results, Triton X-100 may have reduced the storage and loss modulus of MeninGEL owing to its damaging effect on collagen fibrils and sGAG.^4,9^

### 3.6 Gelation Performance of Hydrogels

The mechanical strength and pore diameter of decellularized hydrogels must be compatible with those of the host tissue. Because each tissue is required to resist specific mechanical forces.^54^ The criteria mentioned above may be modified through alterations in collagen concentration within the hydrogel and the cross-linking process.^55^ The gelation images (Figure 3B) demonstrated that all scCO_2_-decellularized samples exhibited successful gelation and hydrogel formation at all three concentrations (5, 7.5, and 10 mg/mL). On the contrary, mature gel formation was not observed in any sample without the neutralization process. Furthermore, hydrogels showed enhanced opacity and toughness postneutralization. Macroscopic observations verified that as the concentration of MeninGELs decreased, their hardness also decreased proportionally.

### 3.7 Biological Evaluations

XTT and live/dead analyses present insight into the cell viability, proliferation, and cytotoxicity in tissue-engineered constructs. Also, the XTT assay quantifies cell metabolic activity associated with cell proliferation. Glioblastoma cells exhibit a high proliferative rate and are typically more stable than other neuronal cells in culture media.^56^ As anticipated, the detergent displayed minimum glioblastoma cell proliferation on day 2 (Figure 3C). However, all specimens showed statistically similar absorbance values at either time point (*n* = 3, Figure 3C). The XTT analysis also quantitatively confirmed that the cell density increased significantly on day 5 relative to that on day 2 in all samples (Figure 3C). Tissue culture plastic (TCP) was used as the control group. The provided live/dead image offers a qualitative assessment of glioblastoma cell viability on MeninGELs (Figure 3D). As illustrated in the figure, the intense green fluorescence indicates the high viability of the cells at both time points (days 2 and day 5). Cell density significantly increased in all groups on day 5 compared with day 2. In addition to this, MeninGEL-200 bar(e+) demonstrated the highest cell viability and dispersion, while detergent(e+) exhibited the lowest at both time points. Consequently, the live/dead and XTT assay outcomes highlighted that the MeninGELs obtained by scCO_2_-decellularization supply an adequate microenvironment for glioblastoma cell growth and proliferation.

## 4 Conclusions

Herein, we investigated the impacts of different methods for the decellularization of bovine spinal cord meninges with scCO_2_. Despite the scCO_2_ successfully penetrating tissue and lysing cells, removing cell fragments from the tissue is insufficient. Studies in the literature generally use scCO_2_/detergent hybrid methods to overcome this problem. These hybrid methods do not entirely eliminate the harsh detergent conditions; instead, they reduce the tissues’ exposure to the detergent. Hence, we focused on combinations of DNase/RNase enzymes with scCO_2_ to eliminate the adverse effects of detergents. The findings indicated that pressures of >180 bar disrupted the surface morphology of the tissue in the scCO_2_ decellularization process. Moreover, our outcomes demonstrate that the 180 bar(e+) method preserved the ECM ultrastructure while simultaneously reducing the dsDNA content by over 95%. According to the compression test, MeninGEL-180 bar(e+) exhibited the highest mechanical strength. Additionally, the decellularization period was reduced from 7 to 3 days with this method. In conclusion, this state-of-the-art technique may be used to decellularize various tissues without detergents and develop biomaterials with tissue-type-specific topologies for clinical tissue engineering applications in the near future.

## Supporting Information Available

Actual image of the scCO_2_ device used in the experiments; images of native and scCO_2_-mediated decellularized tissues; histological intensity analysis graph of Sirius red and Alcian blue staining; and graphics for Young’s modulus calculation of all samples (PDF)
