# Supplementary material for: Supercritical CO2-Mediated Decellularization of Bovine Spinal Cord Meninges: A Comparative Study for Decellularization Performance
Source: ACS Omega. 2024 Nov 25;9(49):48781–90. doi: 10.1021/acsomega.4c08684 (PMC11635505; doi:10.1021/acsomega.4c08684)
Supplement: Supplementary file 1 — ao4c08684_si_001.pdf [file ao4c08684_si_001.pdf]

Supporting Information

Supercritical CO_2-mediated Decellularization of Bovine Spinal Cord Meninges: A Comparative Study for Decellularization Performance

Eren Ozudogru[1], Tugce Kurt[1], Burak Derkus[2], Ugur Cengiz[3, *] and Yavuz Emre Arslan[1, *]

[1]Regenerative Biomaterials Laboratory, Department of Bioengineering, Faculty of Engineering, Canakkale Onsekiz Mart University, Canakkale, Turkey.

[2]Stem Cell Research Laboratory, Department of Chemistry, Faculty of Science, Ankara University, Ankara, Turkey.

[3]Surface Science Research Laboratory, Department of Chemical Engineering, Faculty of Engineering, Canakkale Onsekiz Mart University, Canakkale, Turkey.

*Corresponding authors:

E-mail: yavuzea@gmail.com (Y. E. Arslan)

Tel.: +90-286-218-0018; Fax: +90-286-218-0541

E-mail: ucengiz@comu.edu.tr (U. Cengiz)

Tel.: +90-286-218-0018; Fax: +90-286-218-0541

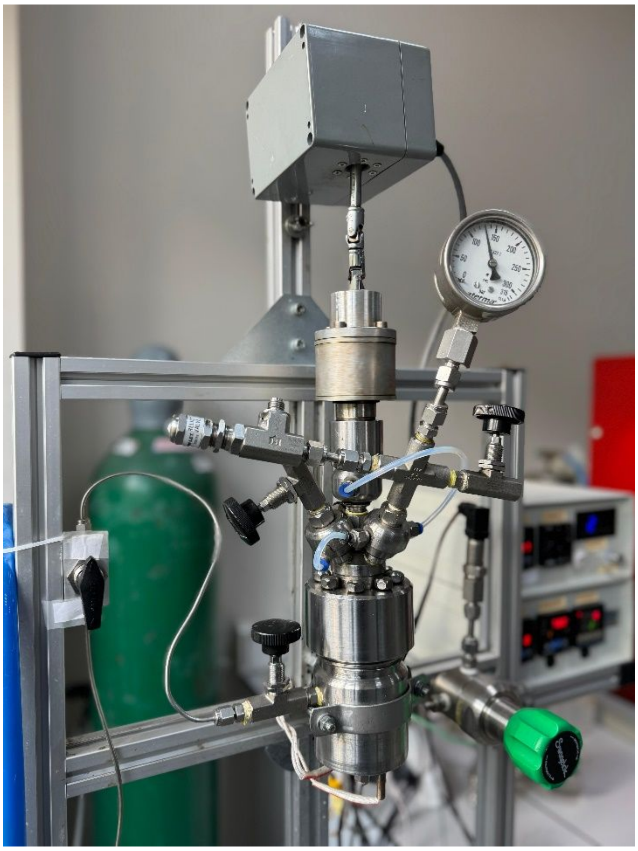

Figure S1. The actual image of the $scCO_2$ system.

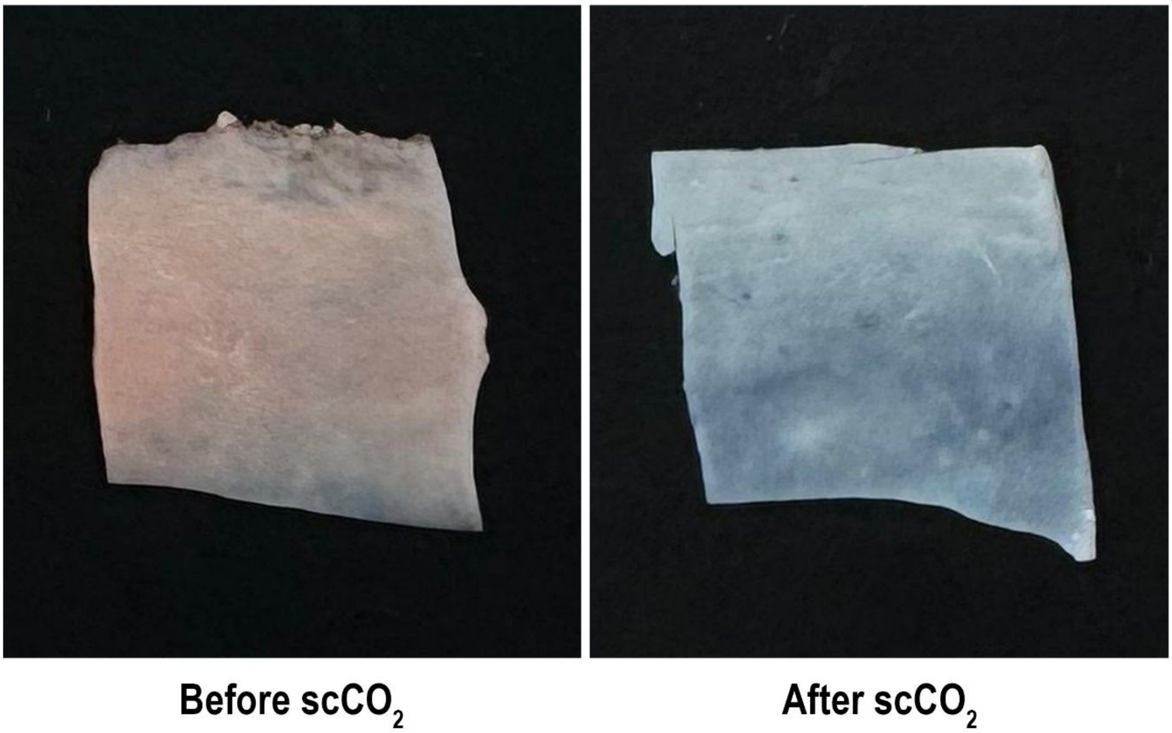

Figure S2. Images of spinal meninges tissue before and after $scCO_2$ atmosphere.

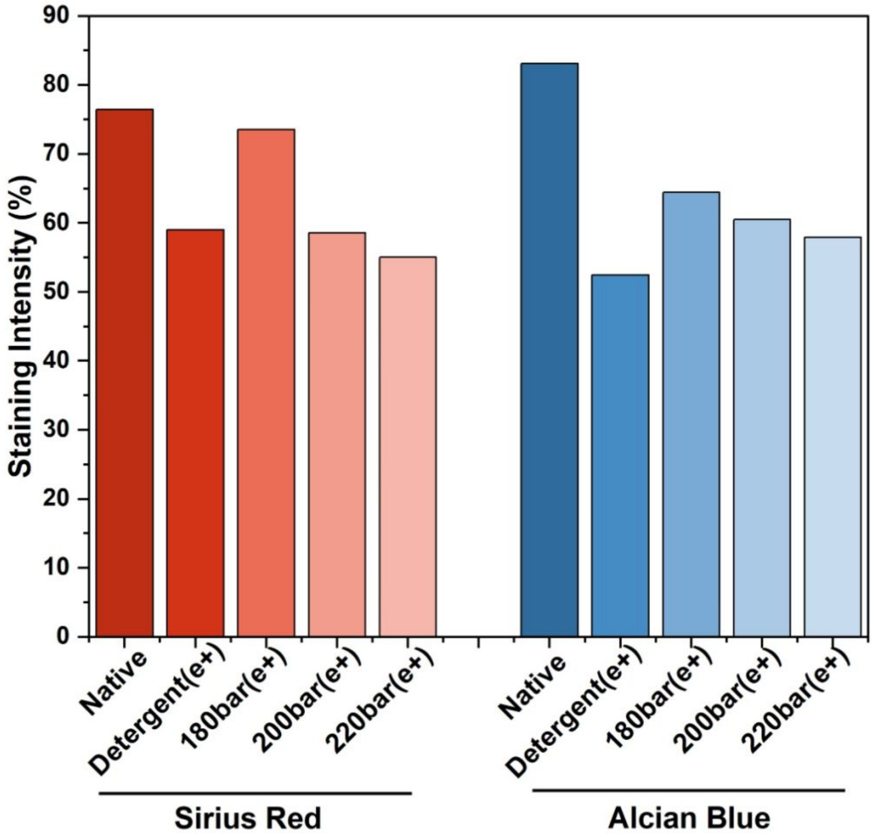

Figure S3. Intensity analysis of Sirius Red and Alcian Blue staining.

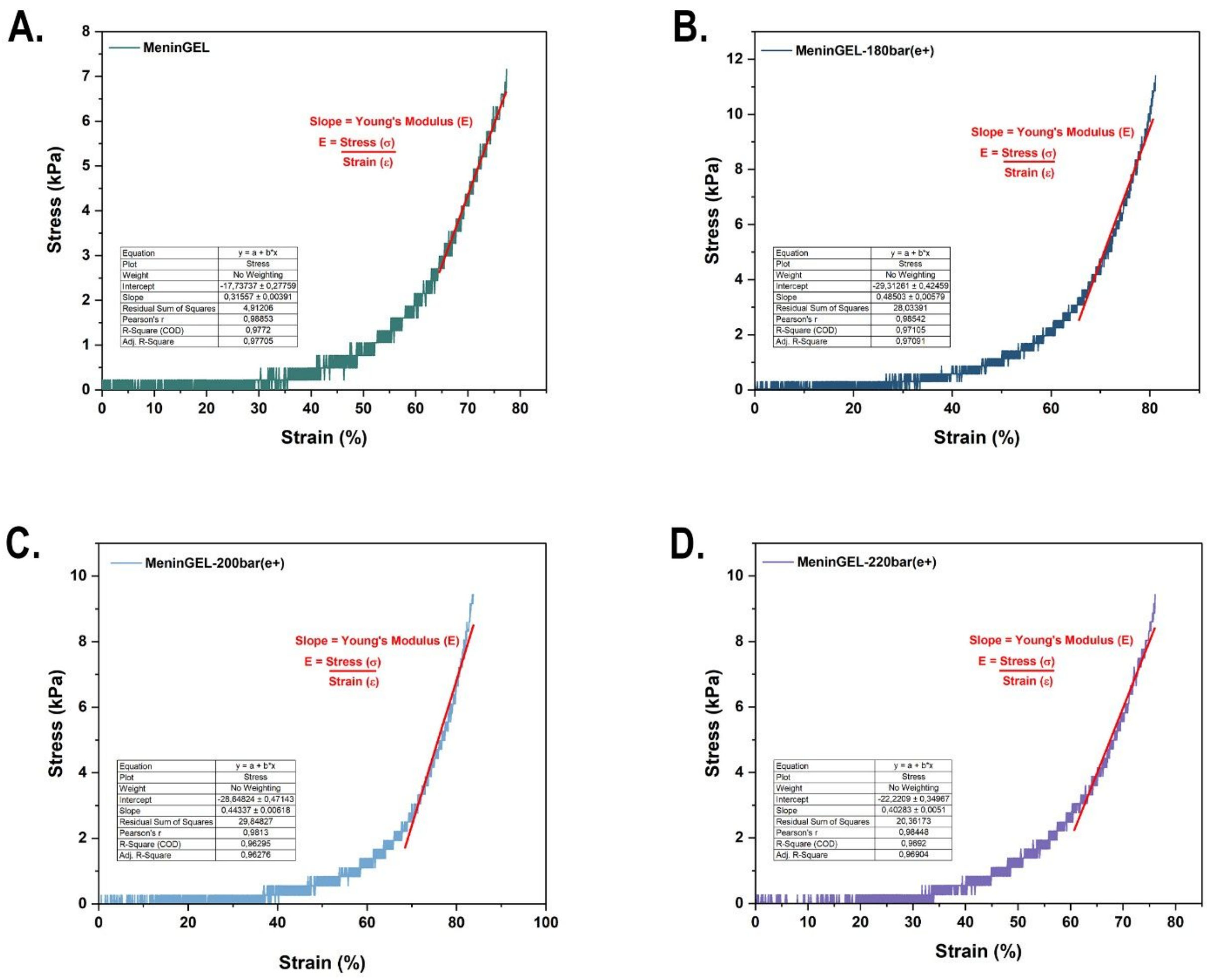

Figure S4. Determination of Young’s modulus with graphical method. MeninGEL (A), MeninGEL-180bar(e+) (B), MeninGEL-200bar(e+) (C), MeninGEL-220bar(e+) (D).
